# Hydrocortisone supresses inflammatory activity of metalloproteinase - 8 in carotid plaque

**DOI:** 10.5935/1678-9741.20150034

**Published:** 2015

**Authors:** Sthefano Atique Gabriel, Leila Antonangelo, Vera Luiza Capelozzi, Camila Baumann Beteli, Otacílio de Camargo Júnior, José Luis Braga de Aquino, Roberto Augusto Caffaro

**Affiliations:** 1 Faculdade de Medicina da Pontifícia Universidade Católica de Campinas (PUC-Campinas), Campinas, SP, Brazil.; 2 Faculdade de Medicina da Universidade de São Paulo (FMUSP), São Paulo, SP, Brazil and Departamento de Citologia do Laboratório Central do Hospital das Clínicas da Faculdade de Medicina da Universidade de São Paulo (HCFMUSP), São Paulo, SP, Brazil.; 3 Faculdade de Medicina da Universidade de São Paulo (FMUSP), São Paulo, SP, Brazil and Departamento de Patologia da Faculdade de Medicina da Universidade de São Paulo (HCFMUSP), São Paulo, SP, Brazil.; 4 Instituto Dante Pazzanese de Cardiologia (IDPC), São Paulo, SP, Brazil.; 5 Faculdade de Ciências Médicas da Santa Casa de Misericórdia de São Paulo (FCMSCSP), São Paulo, SP, Brazil.

**Keywords:** Hydrocortisone, Carotid Stenosis, Inflammation, Endarterectomy, Carotid, Matrix Metalloproteinases

## Abstract

**Objective:**

Matrix metalloproteinases are inflammatory biomarkers involved in carotid plaque instability. Our objective was to analyze the inflammatory activity of plasma and carotid plaque MMP-8 and MMP-9 after intravenous administration of hydrocortisone.

**Methods:**

The study included 22 patients with stenosis ≥ 70% in the carotid artery (11 symptomatic and 11 asymptomatic) who underwent carotid endarterectomy. The patients were divided into two groups: Control Group - hydrocortisone was not administered, and Group 1 - 500 mg intravenous hydrocortisone was administered during anesthetic induction. Plasma levels of MMP-8 and MMP-9 were measured preoperatively (24 hours before carotid endarterectomy) and at 1 hour, 6 hours and 24 hours after carotid endarterectomy. In carotid plaque, tissue levels of MMP-8 and MMP-9 were measured.

**Results:**

Group 1 showed increased serum levels of MMP- 8 (994.28 pg/ml and 408.54 pg/ml, respectively; *P*=0.045) and MMP-9 (106,656.34 and 42,807.69 respectively; *P*=0.014) at 1 hour after carotid endarterectomy compared to the control group. Symptomatic patients in Group 1 exhibited lower tissue concentration of MMP-8 in comparison to the control group (143.89 pg/ml and 1317.36 respectively; P=0.003). There was a correlation between preoperative MMP-9 levels and tissue concentrations of MMP-8 (*P*=0.042) and MMP-9 (*P*=0.019) between symptomatic patients in the control group.

**Conclusion:**

Hydrocortisone reduces the concentration of MMP- 8 in carotid plaque, especially in symptomatic patients. There was an association between systemic and tissue inflammation.

## Abstract (pt)

**Objetivo:**

As metaloproteinases são biomarcadores inflamatórios envolvidos na instabilidade da placa carotídea. O objetivo deste estudo foi analisar a atividade inflamatória da MMP-8 e MMP-9 plasmática e presente na placa carotídea, após administração intravenosa de hidrocortisona.

**Métodos:**

Participaram do estudo 22 pacientes portadores de estenose ≥ 70% em artéria carótida (11 sintomáticos e 11 assintomáticos), submetidos à endarterectomia de artéria carótida. Os pacientes foram divididos em dois grupos: Grupo Controle - não foi administrado hidrocortisona e Grupo 1 - foi administrado 500 mg intravenoso de hidrocortisona durante a indução anestésica. As dosagens plasmáticas de MMP-8 e MMP-9 foram efetuadas no pré-operatório (24 horas antes da endarterectomia de artéria carótida) e em 1 hora, 6 horas e 24 horas após endarterectomia de artéria carótida. Na placa carotídea foram mensurados os níveis teciduais de MMP-8 e MMP-9.

**Resultados:**

O grupo 1 exibiu elevação dos níveis séricos da MMP-8 (994,28 pg/ml e 408,54 pg/ml, respectivamente; P=0.045) e MMP-9 (106.656,34 e 42.807,69, respectivamente; P=0.014) em 1 hora após a endarterectomia de artéria carótida, em relação ao grupo controle. Os pacientes sintomáticos do grupo 1 exibiram menor concentração tecidual de MMP-8, em relação ao grupo controle (143,89 pg/ml e 1317,36, respectivamente; P=0.003). Houve correlação entre os níveis pré-operatórios de MMP-9 e as concentrações teciduais de MMP-8 (P=0.042) e MMP-9 (P=0.019) entre os pacientes sintomáticos do grupo controle.

**Conclusão:**

A hidrocortisona reduz a concentração de MMP-8 na placa carotídea, em especial nos pacientes sintomáticos. Houve associação entre a inflamação sistêmica e a tecidual.

**Table:** 

**Abbreviations, acronyms & symbols**	
CEA	Carotid endarterectomy
EDTA	Ethylenediamine tetraacetic acid
ICA	Internal carotid artery
IL	Interleukin
MMPs	Matrix metalloproteinases
TIA	Transient ischemic attack

## INTRODUCTION

Inflammation plays an important role in the development of atherosclerotic plaque. In addition, many immunocompetent cells, responsible for the production of inflammatory biomarkers, are identified at all stages of the atherosclerotic phenomenon^[1,2]^. The imbalance between pro-inflammatory and anti-inflammatory activity of serum and tissue biomarkers contribute to carotid plaque instability and the occurrence of cerebrovascular events such as transient ischemic attack (TIA) and stroke^[3,4]^.

Increased expression of MMP-8 and MMP-9, which are zinc-dependent regulators of the extracellular matrix, has been demonstrated within carotid plaques^[5,6]^. Overexpression of MMP-8 and MMP-9 stimulates cellular migration, infiltration of T lymphocytes and monocytes in the subendothelium, degradation of the fibrous cap and extracellular matrix, arterial remodeling and intraplaque neoangiogenesis^[5,6]^. Furthermore, it degenerates collagen fibers type I, II, III, IV, V, VII, X and XII and elastin, contributing to carotid plaque rupture and hemorrhage^[5,6]^.

Recent publications suggest that immunomodulatory therapies directed against the inflammatory process in carotid plaques should be developed and tested in order to reduce disease progression^[3,7]^. Glucocorticoids are known for its anti-inflammatory and immunosuppressive properties, reducing the secretion of inflammatory cytokines by monocytes, macrophages and lymphocytes^[8,9]^. Elenkov^[10]^, however, has demonstrated that glucocorticoids are immunomodulatory drugs. The objective of this study was to analyze the inflammatory activity of plasma and carotid plaque MMP-8 and MMP-9, after intravenous administration of hydrocortisone.

## METHODS

### Population

Between October/2012 and September/2013, 22 patients with greater than 70% internal carotid artery (ICA) stenosis were admitted to our center for CEA. The selected patients were 15 (68.18%) men and 7 (31.82%) women, with ages ranging from 50 to 84 years (mean: 69.50±9.09 years). Eleven patients (50%) had experienced a previous neurological event, and 11 (50%) patients had no symptoms. Regarding the contralateral ICA stenosis, 12 (54.54%) patients showed stenosis <50%, 8 (36.36%) exhibited stenosis between 50% to 69% and 2 (9.1%) demonstrated stenosis ≥ 70%.

The inclusion criteria were: asymptomatic and symptomatic patients with ≥ 70% ICA stenosis, with indication for CEA. The exclusion criteria comprised: patients who had already undergone CEA; occlusion or < 70% ICA stenosis; patients admitted for carotid artery stenting (post-CEA restenosis, post-irradiation ICA stenosis, high carotid bifurcation); clinical and/or laboratory suspicion of infection; presence of autoimmune or systemic disease; use of anti-inflammatory or glucocorticoid drugs, chemotherapy treatment or immunossupressants; recent (< 1 month) severe infection or recent (< 1 month) stroke and hypersensitivity or contraindication for the use of hydrocortisone.

The study was approved by the Ethics Committee of the Faculty of Medical Sciences of Santa Casa de São Paulo (Protocol: 108.870) and was performed according to the Guidelines of the World Medical Association's Declaration of Helsinki. All patients gave their full informed consent prior to participating in the study.

### Preoperative period

Baseline data were obtained from clinical records, physical examination, routine laboratory measurement, and from a study protocol filled out by the participating patients, including epidemiological data and cardiovascular risk factors, as summarized in Table 1. Hypertension was defined as a systolic blood pressure ≥ 140 mmHg and/or a diastolic blood pressure ≥ 90 mmHg, or current use of antihypertensive medication at the time of CEA. Diabetes mellitus was diagnosed in patients with fasting blood glucose levels ≥ 126 mg/dL and/or current use of hypoglycemic agents. Smoker was defined as currently smoking or cessation of smoking less than 1 month prior to entering the study. Hypercholesterolemia was defined as a total cholesterol concentration ≥ 200 mg/dL or current use of cholesterol-lowering agents. Abdominal obesity was diagnosed as patient's body mass index ≥ 30 Kg/m^2^.

**Table 1:** Clinical and laboratorial characteristics of the study population.

Variables		Control Group	Hydrocortisone Group	*P*
Age (years)		(11)	(11)	0.718+
		69.09±8.30	69.91±10.20	
Gender	Male	72.70%	63.60%	0.647§
	Female	27.30%	36.40%	
Hypertension	Yes	90.90%	100%	0.306§
	No	9.10%	0%	
Diabetes mellitus	Yes	54.50%	81.80%	0.170§
	No	45.50%	18.20%	
Smoking	Yes	36.40%	63.60%	0.201 §
	No	63.60%	36.40%	
Obesity	Yes	9.10%	27.30%	0.269§
	No	90.90%	72.70%	
BMI (kg/m^2^)		26.64±3.61	26.26±5.14	0.669+
Total Cholesterol (mg/dL)		189.45±22.39	160.09±33.43	0.023+
HDL (mg/dL)		47.55±11.26	41.18±13.56	0.200+
LDL (mg/dL)		110.64±27.23	92.91±27.53	0.212+
Triglycerides (mg/dL)		156.09±50.08	156.91±112.12	0.511+
Glucose (mg/dL)		129.36±53.82	110.27±32.23	0.869+
Carotid cross - clamping (minutes)		44.18±7.22	48.36±12.44	0.430+
Contralateral Carotid Stenosis	< 50%	54.50%	54.50%	0.287§
	50% to 69%	45.50%	27.30%	
	> 70%	0%	18.20%	
Neurologic Symptoms	Symptomatic	54.50%	45.50%	0.670§
	Asymptomatic	45.50%	54.50%	
Ischemic heart disease	Yes	36.40%	63.60%	0.201§
	No	63.60%	36.40%	
Myocardial Revascularization	Yes	36.40%	63.60%	0.201§
	No	63.60%	36.40%	

Data shown as mean ± standard deviation or percentage. BMI=body mass index; HDL=high-density lipoprotein; LDL=low-density lipoprotein;

§ Likelihood Ratio Test;

+ Mann-Whitney Test

The degree of carotid stenosis was determined by duplex ultrasonography investigation. In patients with greater than 70% ICA stenosis, carotid disease was confirmed by cerebral angiography performed up to one month prior to CEA. All patients were examined by a neurologist for assessment of their preoperative neurological status. As observed in previous publications, we followed the North American Symptomatic Carotid Endarterectomy Trial Criteria for classifying patients as being neurologically symptomatic or asymptomatic^[11]^.

Patients submitted to CEA were divided into two groups: a Control group (11 patients) - patients who did not received intravenous hydrocortisone; and a Hydrocortisone group (11 patients) - patients who received a single 500 mg dose of intravenous hydrocortisone during anesthetic induction. Patients' randomization was done with aleatory distribution of 22 sequential numbers in envelopes listed from 1 to 22. Even numbers referred to patiens from the control group and odd numbers referred to patients from the hydrocortisone group. The envelopes were opened in numerical order before anesthetic induction.

### Carotid endarterectomy

CEA was performed under general anesthesia. All endarterectomies were performed by an open, non-eversion technique, with careful surgical exposure of the bifurcation into the internal and external carotid arteries. Patients received 5000 IU of heparin intravenously before cross-clamping. The atheromatous plaque was removed and arteriorrhaphy performed. The mean time of carotid cross-clamping was 46.27±10.16 minutes.

### Hydrocortisone administration

Hydrocortisone sodium succinate (Solu - Cortef^®^) 500 mg, lyophilized in a container, was diluted in 4 ml of distilled water and added to 500 ml of 0.9% saline. The solution was injected intravenously into a peripheral vein at a concentration of 1mg/mL and infusion time of 30 minutes.

### Measurement of serum MMP-8 and MMP-9

Blood samples were obtained via puncture of peripheral veins with needles at four moments: at preoperative (24 hours before CEA) period and at 1 hour, 6 hours and 24 hours after carotid cross-clamping. The blood collected was distributed in two purple tubes containing ethylenediamine tetraacetic acid (EDTA).

The samples were firstly centrifuged at 1.000 rpm for 15 minutes and then at 10.000 rpm for 10 minutes. Plasma was divided into aliquots after sample centrifugation and frozen at -70ºC. The analyses of blood samples (LUMINEX Methodology) collected at different points in time were performed at the Laboratory of Medical Investigation at University of São Paulo. The reference values were: MMP-8 between 350 and 4500 pg/mL and MMP-9 between 3858 and 4050 pg/mL.

### Measurement of MMP-8 and MMP-9 in carotid plaque

After removal, carotid artery plaque was stored at -70ºC for subsequent MMP-8 and MMP-9 measurement (LUMINEX Methodology) at the Laboratory of Medical Investigation at University of São Paulo.

### Statistical analysis

Data were analyzed using the Statistical Package for Social Sciences, version 21.0. Values of continuous variables were expressed as mean ± standard deviation and percentages. Values of *P*<0.05 were considered statistically significant.

For clinical and laboratorial characteristics of the patients, the Likelihood Ratio Test was employed for nonparametric variables and the Mann-Whitney test for parametric variables. The Mann-Whitney test was applied to verify differences between the groups for serum and carotid plaque biomarkers. Correlations between variables were calculated using Spearman rank correlation coefficients.

## RESULTS

### Inflammatory activity of serum MMP-8 and MMP-9 between control group and Hydrocortisone group

In the control group, the concentration of plasma MMP-8 and MMP-9 reduced at 1 hour and 24 hours after CEA and exhibited the highest inflammatory activity at 6 hours (1329.75 pg/ml and 248,583.46 pg/ml respectively) after CEA.

**Fig. 1:**
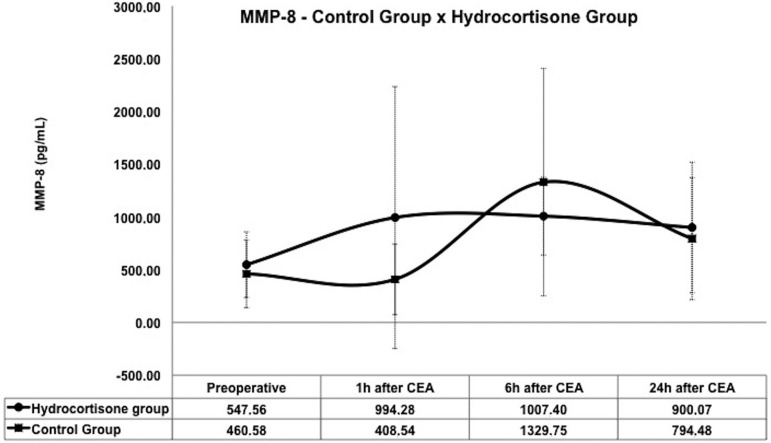
Inflammatory activity of MMP-8 between control group and hydrocortisone group. MMP=metalloproteinase; CEA=carotid endarterectomy

**Fig. 2:**
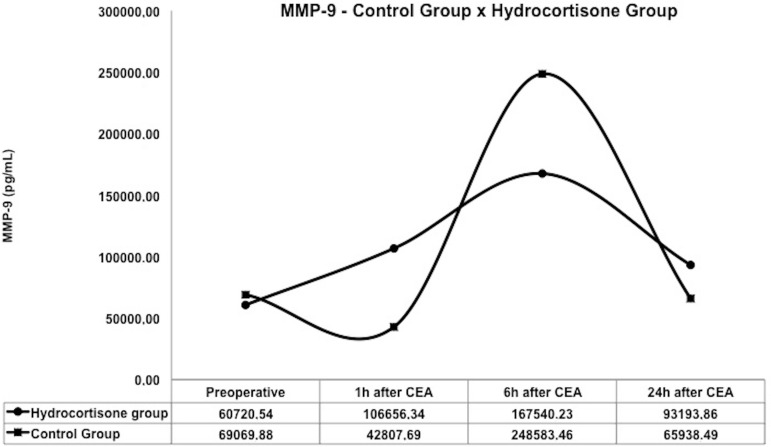
Inflammatory activity of MMP-9 between control group and hydrocortisone group. MMP=metalloproteinase; CEA=carotid endarterectomy

In the hydrocortisone group, a significant increase in serum levels of MMP-8 (994.28 pg/ml and 408.54 pg/ml, respectively; *P*=0.045) and MMP-9 (106,656.34 and 42,807.69 respectively; *P*=0.014) were identified at 1 hour after CEA, while lower concentrations of MMP-8 and MMP-9 were observed at 6 hours after CEA, compared to the control group (Figures 1 and 2).

Table 2 demonstrates the inflammatory activity of plasma MMP-8 and MMP-9, between the control group and hydrocortisone group, during the observation period.

**Table 2:** Inflammatory activity of plasma MMP-8 and MMP-9 between control group and hydrocortisone group.

		Control Group (11)	Hydrocortisone Group (11)	Total (22)	*P*
MMP-8 (pg/ml)	Preoperative	460.58±320.57	547.56±311.03	504.07±311.42	0.412
	1h after CEA	408.54±334.77	994.28±1241.32	701.41±936.47	0.045
	6h after CEA	1329.75±1077.83	1007.40 ± 368.90	1168.57±803.26	0.622
	24h after CEA	794.48±577.92	900.07±619.11	847.27±586.93	0.533
					
MMP-9 (pg/ml)	Preoperative	69069.88±57324.15	60720.54±49210.75	64895.21±52309.05	0.922
	1h after CEA	42807.69±29689.96	106656.34±126215.45	74732.02±95254.03	0,014
	6h after CEA	248583.46±242990.51	167540.23±140492.18	208061.84±198079.88	0.818
	24h after CEA	65938.49±36335.00	93193.86±101289.35	79566.18±75556.14	0.922

Data shown as mean ± standard deviation. MMP=matrix metalloproteinase; CEA=carotid endarterectomy; Mann-Whitney Test

### Inflammatory activity of plasma MMP-8 and MMP-9 between symptomatic patients in the control group and Hydrocortisone Group

Lower concentrations of plasma MMP-8 (890.85 *versus* 1,625.22, respectively; *P*=0.086) and MMP-9 (146,464.50 versus 335,931.02, respectively; *P*=0.153) were observed in symptomatic patients in the hydrocortisone group at 6 hours after CEA, compared to the control group. On the other hand, higher inflammatory activity of plasma MMP-8 and MMP-9 was identified in the hydrocortisone group at 1 hour and 24 hours after CEA, in comparison to the control group.

Table 3 exhibits the inflammatory activity of plasma MMP-8 and MMP-9 between symptomatic patients in the control and hydrocortisone groups, during the follow-up period.

**Table 3:** Inflammatory activity of plasma MMP-8 and MMP-9 between symptomatic patients in control group and hydrocortisone group.

		Control Group (6)	Hydrocortisone Group (7)	Total (13)	*P*
MMP-8 (pg/ml)	Preoperative	621.83±355.76	516.81±328.99	565.28±331.39	0.668
	1h after CEA	466.42±376.86	1134.82±1572.00	826.33±1189.56	0.317
	6h after CEA	1625.22±1025.44	890.85±347.30	1229.79±802.27	0.086
	24h after CEA	983.88±663.43	1093.54±708.74	1042.93±661.66	0.568
					
MMP-9 (pg/ml)	Preoperative	97564.42±65483.60	65284.82±61190.30	80183.10±62764.37	0.317
	1h after CEA	54751.75±32769.94	120945.59±158730.90	90394.59±119268.03	0.153
	6h after CEA	335931.02±263337.80	146464.50±126781.63	233910.59±215861.11	0.153
	24h after CEA	79929.31±20569.79	122526.69±119022.91	102866.36±88023.03	0.668

Data shown as mean ± standard deviation. MMP=matrix metalloproteinase; CEA=carotid endarterectomy; Mann-Whitney Test

### Inflammatory activity of plasma MMP-8 and MMP-9 between asymptomatic patients in control group and Hydrocortisone group

Higher concentrations of plasma MMP-8 and MMP-9 were measured in asymptomatic patients in hydrocortisone group at 1 hour and 6 hours after CEA, while lower serum levels of MMP-8 and MMP-9 were observed in this group at 24 hours after CEA, compared to the control group. There was a significant difference for MMP-9 values, between the hydrocortisone group and the control group, at 1 hour after CEA (81,650.16 pg/ml and 28,474.82, respectively; *P*=0.027).

Table 4 summarizes the inflammatory activity of plasma MMP-8 and MMP-9, between asymptomatic patients in the control and hydrocortisone groups, during the period analyzed.

**Table 4:** Inflammatory activity of plasma MMP-8 and MMP-9 between asymptomatic patients in control group and hydrocortisone group.

		Control Group (5)	Hydrocortisone Group (4)	Total (9)	*P*
MMP-8 (pg/ml)	Preoperative	267.08±113.57	601.38±316.10	415.66±273.79	0.050
	1h after CEA	339.09±302.64	748.33±259.06	520.98±342.76	0.086
	6h after CEA	975.9±1140.96	1211.36±353.88	1080.16±844.60	0.462
	24h after CEA	567.20±407.95	561.49±181.24	564.66±309.09	0.624
					
MMP-9 (pg/ml)	Preoperative	34876.43±13252.10	52733.06±21216.62	42812.71±18579.17	0.142
	1h after CEA	28474.82±19764.07	81650.16±37416.10	52108.30±38803.93	0.027
	6h after CEA	143766.38±189076.18	204422.76±175488.31	170724.77±174486.26	0.327
	24h after CEA	49149.51±46105.68	41861.40±18561.66	45910.35±34739.35	0.624

Data shown as mean ± standard deviation; MMP=matrix metalloproteinase; CEA=carotid endarterectomy; Mann-Whitney Test

### Inflammatory activity of tissue MMP-8 and MMP-9 between symptomatic and asymptomatic patients in control group and Hydrocortisone group

A significant reduction in tissue levels of MMP-8 was observed in symptomatic patients in the hydrocortisone group, compared to the control group (14.89 pg/ml and 1317.36, respectively; *P*=0.003). On the other hand, MMP-9 levels were lower in the control group, in comparison to the hydrocortisone group.

No significant difference was found in tissue concentrations of MMP-8 and MMP-9 between asymptomatic patients in the control and hydrocortisone groups.

Table 5 describes the inflammatory activity of tissue MMP-8 and MMP-9, between asymptomatic and symptomatic patients in the control group and the hydrocortisone group.

**Table 5:** Inflammatory activity of tissue MMP-8 and MMP-9 between asymptomatic and symptomatic patients in control group and hydrocortisone group.

		Control Group (11)	Hydrocortisone Group (11)	TOTAL (22)	*P*
MMP-8 (pg/ml)	Symptomatic	1317.36±1889.05	143.89±62.48	685.49±1363.66	0.003
	Asymptomatic	410.78±283.72	481.59±419.78	442.25±328.21	> 0.999
					
MMP-9 (pg/ml)	Symptomatic	6362.56±5901.41	9810.42±14487.32	8219.10±11074.88	0.886
	Asymptomatic	3584.74±4263.05	2187.64±1858.22	2963.80±3305.13	0.806

Data shown as mean ± standard deviation; MMP=matrix metalloproteinase; CEA=carotid endarterectomy; Mann-Whitney Test

### Correlation between plasma and tissue MMP-8 and MMP-9 in symptomatic patients in control group

An important correlation was observed between preoperative levels of MMP-9 and tissue MMP-8 (Spearman=0.829; *P*=0.042) and MMP-9 (Spearman=0.886; *P*=0.019). Furthermore, a relevant association was identified between tissue MMP-8 and plasma MMP-8 in its highest moment of inflammatory activity (6 hours after EAC) (Spearman=0.886; *P*=0.019).

## DISCUSSION

Although the effect of glucocorticoids on inflammatory activity of metalloproteinases has been explored in the medical literature, to the best of our knowledge, this study demonstrates for the first time that intravenous hydrocortisone may interfere with the inflammatory activity of serum and tissue MMP-8 and MMP-9 in patients with advanced ICA stenosis. Lower concentrations of tissue MMP-8 in symptomatic patients and the tendency to reduce plasma MMP-9 at the moment of its highest activity (6 hours after CEA) demonstrate that the anti-inflammatory property of hydrocortisone reduces the inflammatory activity of MMP-8 and MMP-9 present in peripheral blood and in carotid plaque.

Previous publications have exhibited lower concentrations of serum inflammatory biomarkers using methylprednisolone, but they have not shown this effect on MMP-8 and MMP-9, neither demonstrated this inflammatory reduction with hydrocortisone administration nor evaluated this alteration in biomarkers involved in advanced ICA stenosis and in patients submitted to CEA. After administering 30 mg/kg of methylprednisolone, before surgery and before declamping of thoracic aorta, in 16 patients undergoing elective coronary artery bypass graft, Kawamura et al.^[12]^ observed a significant reduction in concentrations of plasma interleukin (IL) - 6 and IL -8 at 1 hour, 2 hours and 3 hours after declamping of thoracic aorta. Komori et al.^[13]^, after administering 1g of methylprednisolone two hours before elective reconstruction of infrarenal abdominal aortic aneurysms, identified lower concentrations of IL-6 after declamping of abdominal aorta and on the first postoperative day, and reduced levels of C-reactive protein on the first postoperative day, in comparison to patients who had not received a preoperative dose of methylprednisolone. In this study, a single dose of 500 mg hydrocortisone administered during anesthetic induction was chosen since this medication is widely used in the hospital setting. This single dose is known not to cause side effects in our patients and the short half-life of this glucocorticoid (1.5 to 2 hours) may interfere with the inflammatory activity of MMP-8 and MMP-9, which are biomarkers of acute phase response.

The inflammatory response after a surgical procedure involves ischemia-reperfusion injury to the end organs, as a result of arterial crossclamping, and the restoration of perfusion after arterial crossclamping^[14]^. Furthermore, the persistence of any degree of inflammation may be considered potentially harmful to the cardiovascular patient submitted to surgery^[14]^. Rubens & Messana^[15]^ and Liguori et al.^[16]^ have concluded that the systemic inflammatory response is variable and is influenced by comorbidities exhibited by patients, non-pharmacological intervention during surgery, type of anesthesia, perioperative hemodynamic conditions, surgical aspects (surgical incision, duration, time of arterial crossclamping and need for blood transfusion) and postoperative evolution. In this study, we standardized general anesthesia and classical CEA with longitudinal arteriotomy, in order to keep our patients under the same perioperative conditions. The measurement of serum MMP-8 and MMP-9 were performed after carotid declamping in order to evaluate the highest production of these biomarkers during the time of carotid crossclamping. Hydrocortisone, however, was administered during anesthetic induction rather than in the postoperative period in order to evaluate its immunomodulatory effects also upon biomarkers present in carotid plaque.

In our study, symptomatic patients exhibited higher levels of serum MMP-8 and MMP-9 in the preoperative period and in postoperative follow-up, compared to asymptomatic patients. This higher concentration of plasma MMP-8 and MMP-9 in symptomatic patients was also observed by Heider et al.^[17]^; however, his preoperative levels of MMP-8 and MMP-9, both in symptomatic and asymptomatic patients, were higher than those demonstrated in our groups. We believe that this difference has occurred due to the characteristics of the patients analyzed and the methods used for measuring these metalloproteinases.

The postoperative concentrations of MMP-8 and MMP-9, in this study, demonstrated higher inflammatory activity at 6 hours after CEA and a reduction in their levels at 24 hours after CEA. Taurino et al.^[18]^, after evaluating 15 patients undergoing CEA, identified a significant reduction in serum levels of MMP-9 in 46.7% of them in one week and in 93.4% of them in thirty days after CEA. Intraoperative administration of hydrocortisone, however, has shown a tendency to reduce the concentrations of MMP-8 and MMP-9 at 6 hours after CEA, and this tendency has also been observed in symptomatic patients who received a single dose of hydrocortisone. This behavior of MMP-8 and MMP-9 after administration of hydrocortisone may consist in an immunomodulatory effect of hydrocortisone upon circulating macrophages, which are important secretory cells of MMP-8 and MMP-9. In the group in which intravenous hydrocortisone was administered, the non-significant reduction in these biomarkers at 6 hours after CEA can be explained by the small sample size of this study, the preoperative administration of hydrocortisone, and the short half-life of these corticosteroids. Nevertheless, this result suggests a possible interference of hydrocortisone in the inflammatory activity of MMP-8 and MMP-9.

An important result found in this study was the higher concentrations of plasma MMP-8 and MMP-9, at 1 hour after CEA in the hydrocortisone group. We believe that due to the dynamism between the action of inflammatory biomarkers and the activation of systemic defense mechanisms, the presence of hydrocortisone in peripheral blood as an anti-inflammatory agent has stimulated an early compensatory inflammatory activity of MMP-8 and MMP-9 in an attempt to balance the systemic inflammatory and anti-inflammatory response. Therefore, lower inflammatory activity of MMP-8 and MMP-9 at 6 hours after CEA was identified in patients who received a single dose of hydrocortisone, compared to the control group.

As observed in plasma measurement, symptomatic patients exhibited higher concentrations of MMP-8 and MMP-9 in carotid plaque in comparison to asymptomatic patients. We found a significant reduction in inflammatory activity of tissue MMP-8 in symptomatic patients in the hydrocortisone group, demonstrating the capacity of these corticosteroids to interfere also with the inflammatory activity present in carotid plaque. Sluijter et al.^[19]^ demonstrated that tissue MMP-8 and MMP-9 are associated with unstable plaques with lower collagen content, lower fibrous layer and higher risk of rupture. Jiang et al.^[20]^, in an experimental study in pigs, demonstrated an association between intraplaque hemorrhage and an overexpression of MMP-9 present in carotid plaque. Peeters et al.^[21]^, in a follow-up of 3 years after CEA, concluded that higher concentrations of MMP-8 in carotid plaque are associated with higher incidence of coronary events and higher need for peripheral vascular interventions.

The correlation between preoperative levels of MMP-9 and the expression of tissue MMP-9 in symptomatic patients was also observed by Taurino et al.^[18]^. This association suggests the existence of a vicious circle between systemic and tissue inflammation. Alvarez et al.^[22]^ showed a strong association between elevated preoperative levels of MMP-9 and the presence of unstable carotid plaques. In addition, the correlation between tissue MMP-8 and the moment of the highest inflammatory activity of serum MMP-8 at 6 hours after CEA, in symptomatic patients, suggests that inflammation present in carotid plaque may influence systemic inflammation and, as a consequence, maintaining the systemic postoperative inflammatory response even after carotid plaque removal and the restoration of cerebral perfusion.

This study has some limitations. Despite hydrocortisone administration having affected the inflammatory activity of MMP-8 and MMP-9 after CEA and due to the small sample size included, our results do not provide prognostic information regarding the progression of contralateral carotid artery disease and carotid restenosis. Our results, however, demonstrate that intravenous hydrocortisone can reduce inflammatory response associated with CEA, providing a relevant basis for future studies evaluating the effect of intravenous hydrocortisone on the prognosis of patients submitted to CEA.

## CONCLUSION

In conclusion, the immunomodulatory effect of hydrocortisone is identified both in plasma and in carotid plaque, with a significant reduction in the concentration of MMP-8 present in carotid plaque, especially in symptomatic patients, and a tendency to reduce the inflammatory activity of plasma MMP-8 and MMP-9, at the highest postoperative inflammatory response of these biomarkers.

**Table:** 

**Authors’ roles & responsibilities**	
SAG	Analysis and/or interpretation of data; statistical analysis; final approval of the manuscript; study design; implemen-tation of projects and/or experiments; manuscript writing or critical review of its content
LA	Analysis and/or interpretation of data; final approval of the manuscript
VLC	Analysis and/or interpretation of data; final approval of the manuscript
CBB	Analysis and/or interpretation of data; final approval of the manuscript; manuscript writing or critical review of its content
OCJ	Analysis and/or interpretation of data; final approval of the manuscript; study design
JLBA	Analysis and/or interpretation of data; final approval of the manuscript; study design; manuscript writing or critical re-view of its content
RAC	Analysis and/or interpretation of data; final approval of the manuscript; study design; manuscript writing or critical re-view of its content
